# Effectiveness of hypotension prediction index software in reducing intraoperative hypotension in prolonged prone-position spine surgery: a single-center clinical trial

**DOI:** 10.1007/s10877-025-01303-0

**Published:** 2025-05-23

**Authors:** Myrto A. Pilakouta Depaskouale, Stela A. Archonta, Sofia Κ. Moutafidou, Nikolaos A. Paidakakos, Antonia N. Dimakopoulou, Paraskevi K. Matsota

**Affiliations:** 1 2nd Department of Anesthesiology, School of Medicine, National and Kapodistrian University of Athens, “Attikon” Hospital, Athens, Greece; 2 Department of Anesthesiology, Athens General Hospital “Georgios Gennimatas”, Athens, Greece; 3 Department of Neurosurgery, Athens General Hospital “Georgios Gennimatas”, Athens, Greece

**Keywords:** Hypotension prediction index, HPI, Hypotension, Postoperative complications

## Abstract

Intraoperative hypotension (IOH) is associated with morbidity and mortality. The Hypotension Prediction Index (HPI), a machine learning-based tool, offers the opportunity for a proactive approach by predicting hypotensive events. This single center, single blind randomized clinical trial aimed to evaluate the hypothesis that an HPI software-guided approach to IOH management during prone position spine surgery could reduce its incidence compared to our standard care practices. 85 adult patients undergoing spine fusion surgery in the prone position were enrolled. Patients were randomized with a 1:1 allocation ratio. Participants were blinded to their group allocation. In the intervention group, the HPI software was actively used to guide IOH management. In the control group, HPI software readings were blinded, and standard care was administered. The primary outcome was the comparison of time-weighted average (TWA) of IOH between the two groups. Secondary outcomes included a comparison of the incidence of postoperative in-hospital events related to IOH between groups. 77 patients were included in the final analysis (39 in the intervention group), as 8 patients were excluded due to technical issues. No statistically significant difference was found between the intervention and control groups in the TWA of IOH (0.10 mmHg [0.05, 0.23] vs. 0.15 mmHg [0.09, 0.37], p-value 0.088). However, the total duration of hypotensive events per patient was significantly lower in the intervention group (4 min [0.5, 12.2] vs. 11.2 min [2.6, 20.1]; p-value 0.019). Postoperative complication rates did not differ significantly between the two groups. HPI-guided management did not significantly reduce the TWA of IOH compared to standard care in patients undergoing prone-position spine surgery. Complication rates were similar between the two groups.

Clinical Trial Registration: This trial was registered with ClinicalTrials.gov (registration number: NCT05341167).

**Supplementary Information:**

The online version contains supplementary material available at 10.1007/s10877-025-01303-0.

## Introduction

Intraoperative hypotension (IOH) has been associated with acute kidney injury (AKI) [1–4], acute myocardial injury/myocardial injury after surgery(AMI/MINS) and death [5].

Spine surgery in the prone position presents challenges for anesthesiologists, due to prolonged duration, significant blood loss [6] and the hemodynamic effects of prone positioning [7–10]. The prone position can decrease venous return to the heart due to inferior vena cava compression [9] and decrease thoracic compliance, which increases thoracic pressure and reduces left ventricular preload [10]. These challenges can be compounded by perioperative surgical stress response and systemic inflammatory response syndrome (SIRS) [11], which can lead to vasodilation and hypotension [12, 13].

The use of invasive or minimally invasive hemodynamic monitoring in complex spine surgery to assess fluid responsiveness and guide goal-directed fluid therapy (GDFT) is recommended [14]. It is also suggested that baseline blood pressure be used to set individualized intraoperative mean arterial pressure (MAP) targets. However, maintaining an intraoperative MAP above 65 mmHg, may help reduce the risk of AKI and AMI even without baseline MAP consideration [14]. GDFT aims to minimize complications related to fluid imbalance during major surgery, titrating the administration of fluids, vasopressors, and inotropes to achieve hemodynamic targets tailored to the patient’s physiology [15, 16].

IOH is a recognized modifiable factor influencing postoperative complications [2, 17]. Substantial research focuses on its prediction and prevention to improve postoperative outcomes. The Hemosphere monitor with Acumen Hypotension Prediction Index (HPI) software, originally developed by Edwards Lifesciences and, as of September 2024, owned by BD (Becton, Dickinson and Company), is designed to predict IOH by analyzing pulse wave data. It provides an index (0–100) that indicates the likelihood of IOH, along with advanced hemodynamic metrics like cardiac output, contractility, dynamic arterial elastance, and stroke volume variation [18]. A HPI over 85 signals a high risk of an upcoming hypotensive event, prompting preventive management.

Randomized clinical trials (RCTs) [19–27] and metanalyses [28, 29] have shown that using HPI software within a treatment protocol can significantly reduce IOH incidence. However, concerns regarding its accuracy and validation have emerged [30, 31]. Some studies suggest its performance is comparable to setting a MAP alarm at 72–73 mmHg [32–35] while a recent multicenter trial[36] failed to demonstrate a reduction in postoperative AKI in the HPI-guided group.

Given the increased risk of IOH during prone-position spine surgery and the potential of HPI software to mitigate this risk, we undertook an RCT to evaluate the hypothesis whether an HPI guided-intervention would reduce IOH in this specific population and compare the incidence of IOH associated complications.

## Methods

### Study design

This single-center, single-blind, randomized controlled trial was conducted at “Georgios Gennimatas” General Hospital of Athens, a tertiary care facility, between 11 th May 2022 and 4 th November 2023. The study aimed to evaluate whether the use of HPI software accompanied by a specific treatment protocol would reduce IOH compared to a control group during spine surgery lasting more than two hours in the prone position. This study complies with the Consolidated Standards of Reporting Trials checklist[37] and was conducted in accordance with the ethical standards of the Helsinki Declaration.

### Participants

#### Eligibility criteria

Adult patients, over 18 years old, scheduled for posterior thoracic and/or lumbar spinal fusion surgery in the prone position were eligible for inclusion. Patients were excluded if they had a history of severe aortic and/or mitral regurgitation, heart failure with reduced ejection fraction (LVEF < 35%), persistent atrial fibrillation or other significant cardiac arrhythmias, preoperative hypotension [defined as vasopressor requirement to achieve an acceptable MAP (> 65 mmHg) before surgery] or end-stage renal disease on dialysis/renal replacement therapy.

#### Enrollment and recruitment

Patients scheduled for surgery were pre-screened based on their medical records. Eligible patients were approached in the preoperative clinic and provided with detailed information about the study.

### Interventions

#### HPI group

**Fig. 1:**
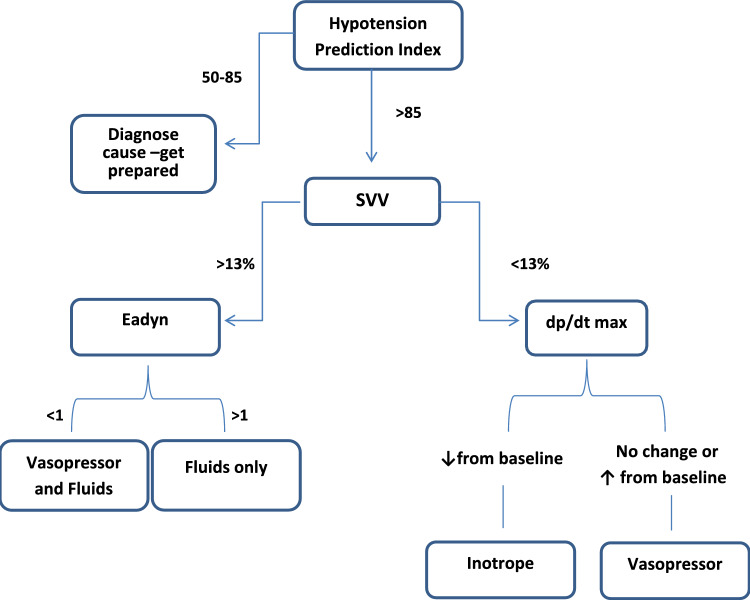
Treatment protocol for the HPI-guided group; SVV, Stroke Volume Variation

In this group, the HPI software was used to prevent hypotensive episodes. The HPI threshold was set at 85, triggering preemptive interventions to maintain MAP above 65 mmHg. The anesthesiologist was required to intervene within two minutes of the alarm, considering the available hemodynamic parameters and following a treatment protocol designed according to current literature [38, 39] (Fig. 1).

#### Control group

In this group the standard, local, anesthetic protocol was provided. Hypotensive episodes were treated with vasoactive agents and fluids. Anesthesiologists were instructed to closely monitor patients and avoid hypotension with MAP < 65 mmHg. HPI algorithm recordings were blinded and unavailable to the anesthesiologist throughout the procedure.

The vasopressor of choice was phenylephrine for bolus administration and norepinephrine for continuous infusion. The inotrope of choice was ephedrine for bolus doses and dobutamine for continuous infusion.

## Protocol details

### Preoperatively

Patient characteristics, medical history, medication usage and the American Society of Anesthesiologists Physical Score Classification (ASA) were documented during the preoperative assessment. In addition, the levels of hemoglobin, hematocrit, urea, creatinine and high sensitivity troponin I(hs-TropI), as well as an electrocardiogram were obtained from their medical records.

### Intraoperatively

All patients received a standardized general anesthesia protocol, including total intravenous anesthesia (TIVA) with target-controlled infusion (TCI) for induction and maintenance using propofol and remifentanil. Depth of anesthesia was monitored via the Patient State Index (PSI) with a target range of 25–50. Neuromuscular blockade was achieved with rocuronium. Volume-controlled ventilation was set at 8 mL/kg of predicted body weight, maintaining End Tidal CO_2_ (ETCO₂) between 30 and 35 mmHg, with a 50:50 O₂/air gas mixture. Quantitative neuromuscular monitoring was applied, and spontaneous breathing activity was suppressed throughout the procedure.

The same monitoring was applied to all participants. This included non-invasive monitoring of blood pressure, SpO2, continuous electrocardiographic monitoring, ETCO2 and urinary output. Additionally, invasive continuous measurement of the patient’s blood pressure was available via radial artery catheterization prior to the induction. The arterial catheter was connected to both the standard monitor and the HemoSphere platform, with data collection from the HemoSphere platform initiated before the administration of any medication. Arterial blood gas testing was performed on an hourly basis.

Intraoperative fluid management for both groups was standardized using a protocol of balanced crystalloid solutions at 4–6 mL/kg/h, with adjustments made according to the patient’s intraoperative needs and at the anesthesiologist’s discretion.

A researcher was present continuously throughout all surgeries in order to record the necessary information, intraoperative incidents and interventions. PSI values, hemodynamic parameters every 10–15 min and urinary output every hour were recorded. Hemodynamic parameters were retrieved from Hemosphere the monitor. The total doses of propofol, opioids/sedatives, fluids and vasoactive agents were also recorded.

### Postoperatively

Blood samples for hs-TropI were collected after surgery and daily for the following three postoperative days [40, 41]. If elevated hs-TropI levels were observed, patients were assessed for symptoms of myocardial ischemia, and a new electrocardiogram (ECG) was performed. A cardiology consultation was obtained as needed. Creatinine levels and urine output were monitored over the first two postoperative days[42], with acute kidney injury evaluated according to the Acute kidney Injury Network (AKIN) classification as abrupt (within 48 h) reduction in kidney function defined as an absolute increase in serum creatinine of more than or equal to 0.3 mg/dL (≥ 26.4 μmol/L), a percentage increase in serum creatinine of more than or equal to 50% (1.5-fold from baseline), or a reduction in urine output (documented oliguria of less than 0.5 mL/kg per hour for more than six hours)[43]. All in-hospital incidents and in-hospital mortality were also documented.

## Outcomes

### Primary outcome

The primary outcome variable of this study is the Time-Weighted Average (TWA) of IOH. TWA of IOH is defined as the area under threshold (AUT) divided by the total duration of the surgery: TWA = (depth of hypotension in mmHg) * (time in minutes spent below a MAP of 65 mmHg)/(total duration of the operation in minutes). We investigated the hypothesis that an HPI-guided intervention would reduce the IOH compared to the control group.

### Secondary outcomes

Comparison of the incidence of in-hospital postoperative complications (AMI,AKI,death) related to IOH between the 2 groups.

### Sample size calculation

A sample size of 70 subjects, 35 in each group, was estimated to be sufficient to detect an effect size of 0.72 or more between groups in reducing TWA of IOH with 85% power and a 5% level of significance based on data from similar studies when the protocol was created [20, 21]. To account for potential exclusions due to unforeseen technical issues or clinical conditions, we prospectively planned to recruit additional participants, resulting in a total enrollment of 85 patients.

Out of those, eight patients were excluded due to technical issues that prevented the collection of data from the Hemosphere monitor for analysis. These exclusions were unrelated to the randomization or allocation process.

**Fig. 2:**
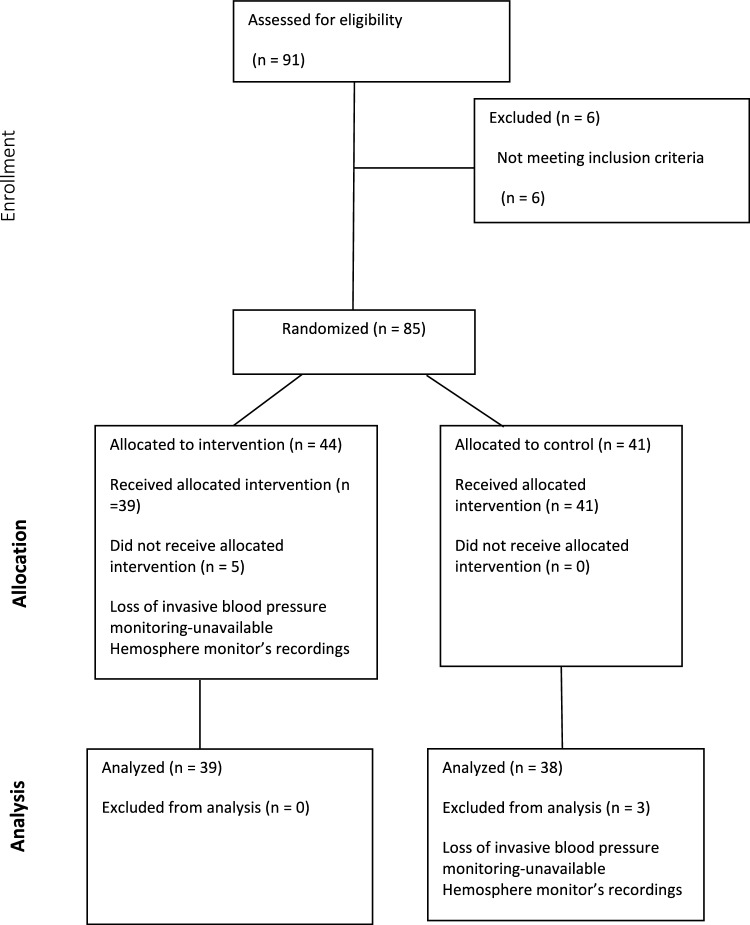
CONSORT Flow Diagram

Ultimately, 77 patients were included in the final analysis, with balanced group sizes maintained as per the original randomization scheme (Fig. 2).

### Randomization

#### Sequence generation

Patients were randomized to either the intervention group or the control group. We used a computer-generated, permuted block randomization with a 1:1 allocation ratio. This resulted in concealed and varying permuted block sizes of either 2 or 4 patients.

#### Allocation concealment

Allocation was concealed using sealed, opaque envelopes prepared by an independent researcher.

#### Implementation

The random sequence was generated by a statistician who had no role in the enrollment process. The anesthesia team opened the envelope immediately before the procedure to assign the participant’s group.

#### Blinding (masking)

The study was single-blind. Although the anesthesia team was aware of the group allocation to allow appropriate response to HPI alerts, participants were blinded to their group assignment. Postoperative outcome assessors were also blinded to the allocation.

### Statistical methods

Continuous variables were presented as mean ± standard deviation (SD) if they followed a normal distribution; otherwise, they were expressed as median and interquartile range (IQR). Categorical variables were summarized using absolute and relative frequencies. Normality was assessed using the Shapiro–Wilk test. Comparisons of proportions were conducted using the Chi-square test or Fisher’s exact test when the assumptions for the Chi-square test were not met. For continuous variables, differences between groups were analyzed using *t* tests if normality was satisfied, and Mann–Whitney *U* test if it was not. P-values for the primary outcome are one sided according to the primary hypothesis. However, since all other similar studies use two-sided testing, we also provide our two-sided analysis in Online Resource 3. All other p-values were two-tailed, and statistical significance was defined as p < 0.05. Data analysis was performed using R version 4.3.1.

## Results

### Baseline demographic and clinical characteristics

Seventy-seven patients were included in the final analysis, with 39 patients in the intervention group and 38 in the control group. Baseline demographic and clinical characteristics are summarized in Table 1. Both groups were comparable, with no significant differences observed. Urgent or emergency cases accounted for 21.1% of patients in the intervention group and 13.2% in the control group. Six patients in each group were classified as ASA III/IV. The most common comorbidity was arterial hypertension, followed by anemia and diabetes mellitus.

**Table 1:** Baseline demographic and clinical characteristics

	Intervention Group (39)	Control Group (38)	p-value
**Baseline characteristics**			
Age^a^ (years)	63 (54, 74)	66 (58, 72)	0.59
Sex^b^			
Male	19 (48.7%)	14 (36.8%)	0.41
Female	20 (51.3%)	24 (63.2%)	
BMI^c^(kg/m^2^)	28.43 (5.3)	28.56 (4.5)	0.91
Preoperative MAP^a^(mmHg)	98 (94, 102)	97 (92, 103)	0.69
ASA classification^b^			
I II III IV	1 (2.6%) 32 (82.1%) 6 (15.4%) 0 (0%)	0 (0%) 32 (84.2%) 5 (13.2%) 1 (2.6%)	0.999*
Etiology of surgery^b^			
Degenerative Redo Trauma Tumor	25 (64.1%) 4 (10.3%) 7 (17.9%) 3 (7.7%)	27 (71.1%) 6 (15.8%) 3 (7.9%) 2 (5.3%)	0.55*
Emergency^b^	8 (21.1%)	5 (13.2%)	0.54
Levels of fusion^a^	3 (2, 4)	2 (1, 4)	0.35
**Pre-existing conditions**^b^			
Arterial hypertension	22 (56.4%)	24 (63.2%)	0.71
Coronary disease	5 (12.8%)	4 (10.5%)	0.999*
Smoking	20 (51.3%)	17 (44.7%)	0.73
Pulmonary disease	9 (23.1%)	6 (15.8%)	0.60
Diabetes mellitus	8 (20.5%)	10 (26.3%)	0.74
Chronic kidney disease	5 (12.8%)	5(13.1%)	0.999*
Neurogenic intermittent claudication/muscle weakness	16 (41%)	17 (44.7%)	0.92
Anaemia	13 (33.3%)	9 (23.7%)	0.49
**Pre-existing medication**^b^			
Beta blockers	9 (23.1%)	8(21.1%)	0.999
ACE inhibitors	7 (17.9%)	3(7.9%)	0.33*
AT1 antagonists	12 (30.8%)	19(50%)	0.14
CCB	9 (23.1%)	12(31.6%)	0.56
Diuretics	7 (17.9%)	15(39.5%)	0.07
Anticoagulants	7 (17.9%)	9 (23.7%)	0.73
Statins	15 (38.5%)	17 (44.7%)	0.74
**Baseline laboratory tests**			
Urea^a^ (mg/dL)	32 (25, 41)	38 (28, 47)	0.09
Creatinine^a^ (mg/dL)	0.7 (0.7, 0.9)	0.8 (0.7, 0.8)	0.77
Hs-TropI^a^ (pg/mL)	2 (1, 5.45)	1.6 (0.7, 3)	0.30
Hb^c^ (g/dL)	13.1 (2.0)	13.3 (1.6)	0.59

*BMI* Body Mass Index, *MAP* Mean Arterial Pressure, *ASA classification* American Society of Anesthesiologists Classification, *ACE inhibitors* Angiotensin-Converting Enzyme Inhibitors, *AT1 antagonists* Angiotensin II Type 1 Receptor Antagonists, *CCB* Calcium Channel Blockers, *Hb* Hemoglobin

a: Continuous parameters are presented as medians (IQR), with p-values derived from the Wilcoxon rank-sum test alongside the Hodges–Lehmann estimator and its 95% confidence interval

b: Categorical parameters are presented as number of patients (percentage), with p-values calculated using the chi-square test; values marked with an asterisk (*) were analyzed using Fisher’s exact test

c: Parameters following a normal distribution are presented as mean (SD), with p-values obtained from the *t* test and mean differences accompanied by their 95% confidence intervals.

Cumulative doses of crystalloids, blood products, vasopressors, sedatives, and analgesics are presented in Table 2, along with operative and monitoring times. No significant differences were observed between the two groups for these parameters. For a mean operative time of 305 min, patients received approximately four liters of crystalloids. Median MAP was 82 mmHg(80,84) for the intervention group and 80 mmHg(78,85) in the control group, with no statistically significant difference between the two groups (p-value 0.312). As an exploratory outcome, the percentage of intraoperative time with HPI > 85 was also analyzed, with a median of 14.4% (4.4,33) in the intervention group and 28.3% (8.5,46) in the control group, though this difference did not reach statistical significance (p = 0.083).

**Table 2:** Cumulative doses of crystalloids, blood products and medications during surgery

	Intervention group Median (IQR)	Control group Median (IQR)	Hodges–Lehman estimation (95% C.I.)	p-value (Wilcox)
Crystalloids(mL)	4000 (3500, 4900)	3985 (3425, 4681)	100 (−400, 560)	0.80
Blood(mL)	250 (0, 500)	250 (0, 500)	0 (0, 230)	0.76
Ephedrine(mg)	15 (3.8, 30)	17.5 (10, 33.8)	0 (− 10, 5)	0.53
Ephedrine(mg/kg)	0.20 (0.06, 0.39)	0.21 (0.11, 0.45)	− 0.03 (− 0.15, 0.07)	0.49
Phenylephrine(mg)	0.20 (0.05, 0.38)	0.25 (0, 0.6)	0 (− 0.20, 0.10)	0.54
Patients in need for norepinephrine(n)*	15(38.5%)	9(23.7%)	–	0.25
Norepinephrine total dose(mcg)**	776 (540, 2252)	1928 (550, 2960)	− 344 (− 1888, 520)	0.25
Propofol total dose (mg)	2560 (2132, 3223)	2455.5(1988,3237)	108 (− 286, 541)	0.62
Propofol (mg/kg/min)	0.085 (0.078, 0.094)	0.086 (0.073, 0.099)	0 (− 0.008,0.008)	0.87
Fentanyl(mcg)	350 (250, 500)	263 (250, 400)	50 (− 0.001, 100)	0.11
Morphine(mg)	10 (10,11)	10 (10,12)	0 (− 0.001, 0.001)	0.62

	Mean (SD)	Mean (SD)	Mean difference (95% CI)	p-value (*t* test)
Operative Time (First Incision to Last Suture) (min)	305 (99)	306 (96)	− 0.8 (− 45.1, 43.6)	0.97
Monitoring time(min)	399 (104)	405 (106)	− 6.2 (− 53.9, 41.6)	0.80

*Categorical parameter presented as number of patients (percentage), with p-values calculated using the chi-square test; **only patients who received norepinephrine were analyzed

#### Primary outcome

No statistically significant difference was observed between the two groups in the TWA of the AUT for MAP < 65 mmHg per patient (0.10 [0.05, 0.23] vs. 0.15 [0.09, 0.37], p-value 0.088) in the intervention and control groups respectively (Table 3).

**Table 3:** Cumulative blood pressure parameters

	Intervention group(39) Median (IQR)	Control Group (38) Median (IQR)	Hodges–Lehman estimation (95% C.I.)	p-value (Wilcox)
**MAP < 65 mmHg (primary outcome)**				
Total number of hypotensive events	2 (1, 5)	4 (1, 7)	− 1 (− Inf, − 0.001)	**0.030**
Average duration of each hypotensive event(min)	2 (0.5, 3.4)	2.4 (1.7, 3.3)	− 0.38 (− Inf, 0.06)	0.095
Total duration of hypotensive events per patient(min)	4 (0.5, 12.2)	11.2 (2.6, 20.1)	− 4 (− Inf, − 0.66)	**0.019**
Mean MAP < 65 mmHg per patient (mmHg)	60 (58, 61)	61 (60, 61)	− 0.86(− Inf, 0.04)	0.058
AUT MAP < 65 mmHg for per patient (mmHg*min)	47.33 (24, 98.33)	64.33 (32.67,114.33)	− 13.33(− Inf, 9.33)	0.131
TWA of AUT (MAP < 65 mmHg) per patient (mmHg)	0.10 (0.05, 0.23)	0.15 (0.09, 0.37)	− 0.04(− Inf, 0.01)	0.088
**Postinduction MAP < 65 mmHg**				
Total number of hypotensive events	1 (0, 2)	1 (0, 2)	− 0.001 (− Inf, − 0.001)	**0.026**
Average duration of each hypotensive event (min)	1 (0, 2.3)	2.3 (0.3, 3.8)	− 1 (− Inf, − 0.001)	**0.012**
Total duration of hypotensive events per patient (min)	1 (0, 3.8)	4 (0.3, 5.7)	− 1.33 (− Inf, – 0.001)	**0.010**
Mean MAP < 65 mmHg per patient (mmHg)*	59 (58, 61)	59.32 (58, 61)	0.01 (− Inf, 1.37)	0.505
AUT MAP < 65 mmHg for per patient (mmHg*min)	22.85 (8.92, 50.67)	27.67(13.58,53.50)	− 4.14 (− Inf,8)	0.279
TWA of AUT (MAP < 65 mmHg) per patient (mmHg)	1.14 (0.27, 2.06)	1.34 (0.65,2.33)	− 0.23 (− Inf, 0.36)	0.29
**Operative Time (First Incision to Last Suture) MAP < 65 mmHg**				
Total number of hypotensive events	0 (0, 2)	0 (0, 4)	− 0.001 (− Inf, 0.001)	0.097
Average duration of each hypotensive event (min)	0 (0, 1.5)	0 (0, 2.1)	− 0.001 (− Inf, 0.001)	0.187
Total duration of hypotensive events per patient (min)	0 (0, 3.7)	0 (0, 11.3)	− 0.001 (− Inf, 0.001)	0.102
Mean MAP < 65 mmHg per patient (mmHg)	59 (58, 61)	59 (58, 61)	0.01 (− Inf, 1.37)	0.505
AUT MAP < 65 mmHg for per patient (mmHg*min)	26.17 (15.92,55.25)	37.33 (28,74.33)	− 13.34 (− Inf, 6.34)	0.146
TWA of AUT (MAP < 65 mmHg) per patient (mmHg)	0.08 (0.03,0.17)	0.12 (0.08, 0.36)	− 0.05 (− Inf, 0.01)	0.101
**MAP > 100 mmHg(hypertension)**				
Total number of hypertensive events	5 (3, 9)	3 (1, 7)	1 (− 1,3)	0.2177
Average duration of each hypertensive event > 100 mmHg (min)	3.3 (2.2, 4.2)	3 (1.8, 4.3)	0.17 (− 0.67, 1)	0.683
Total duration of hypertensive events per patient (min)	13 (7.2, 34.2)	10.5 (4.1, 25.9)	3.34 (− 3, 9.67)	0.296
AUT MAP > 100 mmHg for per patient (mmHg*min)	207.33 (76.67, 374.17)	135.34 (40.50, 427.25)	29.17 (− 48, 108.34)	0.558
TWA of AUT (MAP > 100 mmHg) per patient (mmHg)	0.53 (0.20, 0.97)	0.42 (0.10, 0.93)	0.074 (− 0.129, 0.283)	0.482

*MAP* mean arterial pressure, *AUT* area under threshold, *TWA* time weighted average

For the primary outcome measures and measures related to the reduction of IOH tests are one sided; *t test

The only parameters of hypotension showing a statistically significant difference between the two groups was the total number of hypotensive episodes with a difference of medians of −1 episode (p-value 0.03) and the total duration of hypotensive events per patient, with a median of 4 min [0.5, 12.2] in the intervention group compared to 11.2 min [2.6, 20.1] in the control group. This corresponded to a difference of medians of − 4 min [− inf, − 0.66] with a p-value of 0.019(Table 3). An analysis of IOH between the two groups for an additional threshold of MAP < 50 mmHg is provided in Online Resource 1.

There was also no statistically significant difference between the two groups in parameters of hypertension, defined as MAP > 100 mmHg sustained for ≥ 1 min (Table 3).

Postoperative parameters are summarized in Table 4. Additional graphs for lactate, creatinine and hs-TropI levels are given in Online Resource 2. MINS was defined as hs-TropI levels exceeding the local 99 th percentile (< 15.6 pg/mL), with myocardial infarction(MI) further characterized by the presence of clinical symptoms or new ECG findings consistent with infarction [44]. Pulmonary infection was defined by the presence of a fever > 37.8 °C alongside compatible clinical or radiological findings. Postoperative hemodynamic instability was identified as the need for norepinephrine infusion after discharge from the post-anesthesia care unit. Delirium was assessed using the 3D-CAM tool during the first three postoperative days while patients remained hospitalized [45]. No statistically significant differences were observed in the complication rates between the two groups.

**Table 4:** Postoperative parameters

	Intervention group (39)	Control Group (38)	p-value
	Number of events (%)	Number of events (%)	
MINS**	6 (15.8%)	8 (21.1%)	0.77
MI	1 (2.6%)	2 (5.3%)	0.62*
AKIN**			
Stage 1	1 (2.6%)	3 (7.9%)	0.47*
Stage 2	3 (7.9%)	1 (2.6%)	
HDU/ICU stay	7 (17.9%)	3 (7.9%)	0.31*
Pulmonary infection	9 (23.1%)	3 (7.9%)	0.11*
Pulmonary embolism	1 (2.6%)	1 (2.6%)	0.999*
Pleural effusion	0 (0%)	1 (2.6%)	0.49*
Postoperative hemodynamic instability	7 (17.9%)	6 (15.8%)	0.999*
Lac > 2	6 (15.4%)	7 (18.4%)	0.73
Anemia that needs transfusion**	12 (31.6%)	12 (31.6%)	1*
Thrombopenia	10 (25.6%)	7 (18.4%)	0.62
Fever**	14 (36.8%)	10 (26.3%)	0.46
Reoperation	3 (7.7%)	2 (5.3%)	0.999*
Surgical Site Complications	2 (5.1%)	2 (5.3%)	0.999*
Delirium	3 (7.7%)	1 (2.6%)	0.62*
Cardiac arrest/death	0 (0%)	1 (2.6%)	0.49
Length of stay^a^	5 (3, 6)	4 (3, 6)	0.82

*MINS* myocardial injury after surgery, *MI* myocardial infarction, *AKIN* Acute kidney Injury Network, *HDU* high dependency unit, *ICU* intensive care unit, *lac* lactat; all Categorical parameters are presented as number of events (percentage), with p-values calculated using the chi-square test; values marked with an asterisk (*) were analyzed using Fisher’s exact test; (**)missing data from one patient in each parameter; a: Continuous parameter presented as median (IQR), with p-value derived from the Wilcoxon rank-sum test

## Discussion

Our study demonstrated that the use of HPI software accompanied by a treatment protocol did not statistically significantly reduce the TWA of IOH compared to the control group. However, the total duration of hypotensive events per patient was significantly shorter in the intervention group with a median difference of − 4 min (− Inf, − 0.66). Notably in a post-hoc analysis, the intervention group showed shorter average duration and total duration during the postinduction period for each patient. No statistically significant differences were observed between the groups in parameters related to intraoperative hypertension. Postoperative complications related to IOH (AKI, MINS, and MI) did not differ statistically significantly between the two groups. Similarly, the overall rates of in hospital postoperative complications showed no statistically significant differences between the intervention and control groups.

Our findings differ from a recent meta-analysis of seven RCTs [20–24, 26, 29, 46], which demonstrated a statistically significant reduction in the TWA of IOH favoring HPI guidance, with an overall median difference of − 0.21 mmHg (95% CI − 0.33, − 0.09; p-value 0.001). Additionally a recent RCT [27] and 5 cohort studies [47–51] reported similar results. Variations in study designs, inclusion and exclusion criteria, and the types of surgeries, may explain these differences. Regarding the total duration of hypotension per patient, our results are consistent with most of the existing studies and is in agreement with the aforementioned meta-analysis [29] which reported an overall median difference of − 10.11 min (95% CI: − 15.82, − 4.40; p-value 0.001) in favor of the HPI-guided groups.

Our findings align with two existing RCTs [25, 46] that reported no statistically significant reduction in the incidence of IOH with HPI guidance. In one of these studies [46] similar to our own, anesthetists in both groups were instructed to actively avoid hypotension. This approach may have heightened vigilance in the control group, leading to comparable IOH outcomes between the intervention and control groups. Notably, the TWA of IOH for our control group, 0.14 mmHg (0.03–0.39), closely mirrors that reported by Maheshwari et al. [46] and is lower than control groups in other studies [20–23, 26, 27]. This emphasizes the importance of close monitoring of arterial blood pressure in mitigating IOH. These findings suggest that the efficacy of HPI-guided management in reducing IOH may depend on the baseline incidence of intraoperative hypotension in the specific clinical setting [52].

Five studies [20, 21, 23, 27, 50] explored the incidence of hypotension overtreatment in HPI-guided groups. Among these, only two reported statistically but not clinically significant differences in hypertension parameters, noting higher rates of hypertension in the HPI-guided group. We observed no statistically significant differences between the groups in intraoperative hypertension (MAP > 100 mmHg). This consistency across studies may reflect clinicians’ awareness of the potential harm of hypertension, prompting active intraoperative management to mitigate it.

Limited evidence exists for differences in postoperative outcomes related to IOH between patients under HPI guided interventions and control groups. In fact four RCTs [21, 24, 27, 46] and one cohort study [49] reported no differences in AKI incidents whereas three RCTs [21, 27, 46] detected no differences in AMI incidents. This gap may be attributed to the fact that already existing studies are not sufficiently powered to detect such differences. A recently published multicenter RCT [36], powered to detect a meaningful difference on the postoperative complication rate, concluded that the HPI-guided hemodynamic therapy did not reduce the incidence of postoperative AKI or overall complications compared to standard care. However, this study provides no hemodynamic data. In our study, the incidence of MINS was 15.8% in the intervention group and 21.1% in the control group (p-value 0.767). Similarly, the incidence of AKI was approximately 10% in both groups (p-value 0.466). The incidence of MINS in our study appears higher than that reported by Kouz et al. [53] who observed an incidence of 3% within three days of surgery. This discrepancy may be attributed to the types of surgeries included, as our study exclusively involved major procedures with an increased risk of bleeding and higher estimated blood loss, which could impact oxygen delivery.

Most existing studies, including ours, use the TWA of IOH < 65 mmHg as their primary outcome, reflecting the current understanding in the literature that both the severity and duration of hypotension [2, 5, 54, 55] significantly influence postoperative adverse events. This metric enables comparisons across studies on IOH, regardless of surgical duration. A large retrospective cohort analysis [2] reported that baseline factors were more strongly associated with myocardial and renal injury than intraoperative blood pressure; however, blood pressure remains a modifiable factor [2, 17].The authors also highlighted that AMI and AKI were more strongly linked to extreme hypotensive excursions than to overall mean values. They proposed that primary exposure should focus on the lowest MAP sustained for a cumulative 5 min. Another study [42] found that the risk of both AKI and myocardial injury increased substantially at MAPs below 55–60 mmHg, with even brief durations at MAP < 55 mmHg being associated with adverse outcomes. However, consistent with the findings of Ahuja et al. [2] the observed associations were moderate, suggesting the possibility of an underlying biological effect contributing to these outcomes. Sun et al. [56] also concluded that postoperative AKI is associated with prolonged intraoperative periods of MAP less than 60 mmHg. Notably, even though the hypotensive burden in our study was lower in both the control and intervention groups compared to these thresholds, some patients still experienced relevant postoperative complications, suggesting that factors beyond IOH modification may play a role in the lack of observed differences in postoperative outcomes. Similarly another RCT [57] demonstrated that the relationship between intraoperative hypotension and postoperative major adverse cardiac events (MACE) is likely more complex than previously assumed. Despite achieving a 60% reduction in the duration of IOH with MAP < 65 mmHg, the study found no significant reduction in AMI or 30-day MACE/AKI. A similar conclusion was drawn from POISE-3 trial [58] which found that applying different intraoperative MAP thresholds did not significantly impact perioperative hemodynamics or vascular complications. Given the established, albeit moderate, association between MINS and AKI with IOH, it may be worth reconsidering the thresholds analyzed. Most studies report either no or very minimal occurrences of IOH with MAP < 55 or < 50 mmHg, or durations exceeding 15 min. This scarcity of severe or prolonged hypotension might explain why lowering IOH in the HPI-guided group does not result in significant differences in outcomes. Another important factor to consider when evaluating the absence of differences despite reducing IOH is the potential impact of ward hypotension, which often remains underdiagnosed and untreated and may persist for longer durations [17], as demonstrated by a sub-analysis of the POISE-2 trial [59].

Concerning the lack of observed postoperative clinical differences between the HPI-guided and control groups, it is worth considering the ongoing discussion regarding the original validation of the HPI [30, 31, 60, 61] and its predicting ability. Some studies argue that there is a strong correlation between HPI and MAP [32, 34, 35, 62] suggesting that setting a higher MAP alarm threshold, such as 71 mmHg, may be a safe approach to prevent IOH [31]. Ranucci et al. [63] further demonstrated that while the HPI < 85 had a clinically acceptable negative predictive value of 97.8%, its positive predictive value was notably low at 12.6%, making it insufficient as a sole trigger for hemodynamic interventions. This could explain why, in our study, anesthetists in the control group, who were instructed to prevent hypotension, were able to achieve this effectively by monitoring MAP alone.

A key strength of our study is the inclusion of emergency surgeries, unlike most previous studies, which enhances the generalizability of our findings to this patient population. Another notable strength is the insertion of the arterial catheter prior to the induction of anesthesia. The post-hoc analysis of post-induction hypotension revealed a statistically significant difference between the two groups. To date, only one other study [23] has performed a similar analysis, demonstrating that post-induction hypotension was significantly reduced in the HPI-guided group. This observation could suggest that the use of HPI software may be effective in mitigating hypotensive episodes, even when these are primarily attributable to anesthetic techniques. Another strength of our study is the active investigation of all patients for MINS and AKI, even in the absence of symptoms. MINS often remains undetected due to its asymptomatic nature, despite its significant impact on patient prognosis [17, 41]. To our knowledge only two other studies [36, 46] have similarly investigated MINS in the context of HPI-guided management. These findings highlight the influence of undetected factors on this complication, as its incidence was not reduced despite the low levels of IOH.

Several limitations should be considered when interpreting our findings. First, as a single-center study, standard care reflects our local protocol, which may limit the generalizability of the results. Second, the study was underpowered to detect differences in postoperative complications, as the sample size was determined to assess differences in the TWA of IOH. Third, as a single-blind RCT, anesthetists in the control group may have adopted a more vigilant approach than usual practice. Nevertheless, both groups followed an explicit protocol to prevent hypotension. The protocol was developed by our institution based on the existing literature at the time but was not externally validated. Therefore, we cannot exclude the possibility that there may be a more optimal approach to interpreting the available hemodynamic data. Additionally, we did not record every intervention made in response to alarms, raising the possibility that some alarms were ignored in the intervention group. However, given the overall low levels of IOH, we believe this had minimal clinical impact. The low proportion of ASA III/IV patients in our sample also makes it challenging to draw definitive conclusions about the intervention’s potential benefits in higher-risk populations. Missing data can introduce bias, as approximately 10% of randomized patients were subsequently excluded. The reason for missing data was the loss of the arterial catheter during surgery, compounded by the difficulty of timely re-insertion due to the patient being covered with sheets in the prone position. Lastly, all patients were in the prone position, which introduces unique hemodynamic alterations, making it necessary to consider this factor when extrapolating these findings to other surgical settings.

Our findings neither support nor oppose the use of HPI-guided management for IOH. However, we strongly advocate for the scientific focus on IOH and its association with postoperative complications to extend beyond the intraoperative period. Future studies should not only be sufficiently powered to detect clinically meaningful reductions in hypoperfusion-related complications but also include monitoring of postoperative hemodynamic parameters.

In conclusion, HPI guidance did not result in a statistically significant reduction in the TWA of IOH compared to standard care in adult patients undergoing spine surgery in the prone position. Furthermore, no significant differences in postoperative complications were observed between the groups. Further research is essential to define effective strategies for managing modifiable factors that contribute to perfusion-related postoperative complications.

## Supplementary Information

Below is the link to the electronic supplementary material.

Supplementary file1 (PDF 582 KB)

Supplementary file2 (PDF 676 KB)

Supplementary file3 (PDF 276 KB)

## Data Availability

Data collection form is available upon reasonable request.

## Declarations

### Study protocol

Study protocol is available upon reasonable request.

## References

[1] Kim B, Sangha G, Singh A, Bohringer C. The effect of intraoperative hypotension on postoperative renal function. Curr Anesthesiol Rep. 2023;13:181–6. 10.1007/s40140-023-00564-2. 39802614 10.1007/s40140-023-00564-2 PMC11721893

[2] Ahuja S, Mascha EJ, Yang D, et al. Associations of intraoperative radial arterial systolic, diastolic, mean, and pulse pressures with myocardial and acute kidney injury after noncardiac surgery. Anesthesiology. 2020;132:291–306. 10.1097/ALN.0000000000003048. 31939844 10.1097/ALN.0000000000003048

[3] Tu M, Hong S, Lu J, et al. Effect of strict intraoperative blood pressure management strategy on postoperative acute kidney injury in non-cardiac surgery: a meta-analysis of randomised controlled trials. Int J Clin Pract. 2021. 10.1111/ijcp.14570. 34165855 10.1111/ijcp.14570

[4] Shaw AD, Khanna AK, Smischney NJ, et al. Intraoperative hypotension is associated with persistent acute kidney disease after noncardiac surgery: a multicentre cohort study. Br J Anaesth. 2022;129:13–21. 10.1016/j.bja.2022.03.027. 35595549 10.1016/j.bja.2022.03.027

[5] Wesselink EM, Kappen TH, Torn HM, et al. Intraoperative hypotension and the risk of postoperative adverse outcomes: a systematic review. Br J Anaesth. 2018;121:706–21. 10.1016/j.bja.2018.04.036. 30236233 10.1016/j.bja.2018.04.036

[6] Carabini LM, Koski TR, Bebawy JF. Perioperative management for complex spine fusion surgery. Anesthesiology. 2024;140:293–303. 10.1097/ALN.0000000000004744. 38048486 10.1097/ALN.0000000000004744

[7] Yokoyama M, Ueda W, Hirakawa M, Yamamoto H. Hemodynamic effect of the prone position during anesthesia. Acta Anaesthesiol Scand. 1991;35:741–4. 10.1111/j.1399-6576.1991.tb03382.x. 1763593 10.1111/j.1399-6576.1991.tb03382.x

[8] Wadsworth R, Anderton JM, Vohra A. The effect of four different surgical prone positions on cardiovascular parameters in healthy volunteers. Anaesthesia. 1996;51:819–22. 10.1111/j.1365-2044.1996.tb12608.x. 8882241 10.1111/j.1365-2044.1996.tb12608.x

[9] Edgcombe H, Carter K, Yarrow S. Anaesthesia in the prone position. Br J Anaesth. 2008;100:165–83. 10.1093/bja/aem380. 18211991 10.1093/bja/aem380

[10] Kwee MM, Ho Y-H, Rozen WM. The prone position during surgery and its complications: a systematic review and evidence-based guidelines. Int Surg. 2015;100:292–303. 10.9738/INTSURG-D-13-00256.1. 25692433 10.9738/INTSURG-D-13-00256.1 PMC4337445

[11] Cusack B, Buggy DJ. Anaesthesia, analgesia, and the surgical stress response. BJA Education. 2020;20:321–8. 10.1016/j.bjae.2020.04.006. 33456967 10.1016/j.bjae.2020.04.006 PMC7807970

[12] Margraf A, Ludwig N, Zarbock A, Rossaint J. Systemic inflammatory response syndrome after surgery: mechanisms and protection. Anesth Analg. 2020;131:1693–707. 10.1213/ANE.0000000000005175. 33186158 10.1213/ANE.0000000000005175

[13] Lambden S, Creagh-Brown BC, Hunt J, et al. Definitions and pathophysiology of vasoplegic shock. Crit Care. 2018;22:174. 10.1186/s13054-018-2102-1. 29980217 10.1186/s13054-018-2102-1 PMC6035427

[14] Salmasi V, Maheshwari K, Yang D, et al. Relationship between intraoperative hypotension, defined by either reduction from baseline or absolute thresholds, and acute kidney and myocardial injury after noncardiac surgery. Anesthesiology. 2017;126:47–65. 10.1097/ALN.0000000000001432. 27792044 10.1097/ALN.0000000000001432

[15] Blacker SN, Vincent A, Burbridge M, et al. Perioperative care of patients undergoing major complex spinal instrumentation surgery: clinical practice guidelines from the society for neuroscience in anesthesiology and critical care. J Neurosurg Anesthesiol. 2022;34:257–76. 10.1097/ANA.0000000000000799. 34483301 10.1097/ANA.0000000000000799

[16] Ongaigui C, Fiorda-Diaz J, Dada O, et al. Intraoperative fluid management in patients undergoing spine surgery: a narrative review. Front Surg. 2020;7:45. 10.3389/fsurg.2020.00045. 32850944 10.3389/fsurg.2020.00045 PMC7403195

[17] Sessler DI, Khanna AK. Perioperative myocardial injury and the contribution of hypotension. Intensive Care Med. 2018;44:811–22. 10.1007/s00134-018-5224-7. 29868971 10.1007/s00134-018-5224-7

[18] Hatib F, Jian Z, Buddi S, et al. Machine-learning algorithm to predict hypotension based on high-fidelity arterial pressure waveform analysis. Anesthesiology. 2018;129:663–74. 10.1097/ALN.0000000000002300. 29894315 10.1097/ALN.0000000000002300

[19] Schneck E, Schulte D, Habig L, et al. Hypotension Prediction Index based protocolized haemodynamic management reduces the incidence and duration of intraoperative hypotension in primary total hip arthroplasty: a single centre feasibility randomised blinded prospective interventional trial. J Clin Monit Comput. 2020;34:1149–58. 10.1007/s10877-019-00433-6. 31784852 10.1007/s10877-019-00433-6

[20] Wijnberge M, Geerts BF, Hol L, et al. Effect of a machine learning-derived early warning system for intraoperative hypotension vs standard care on depth and duration of intraoperative hypotension during elective noncardiac surgery: the HYPE randomized clinical trial. JAMA. 2020;323:1052. 10.1001/jama.2020.0592. 32065827 10.1001/jama.2020.0592 PMC7078808

[21] Tsoumpa M, Kyttari A, Matiatou S, et al. The use of the hypotension prediction index integrated in an algorithm of goal directed hemodynamic treatment during moderate and high-risk surgery. JCM. 2021;10:5884. 10.3390/jcm10245884. 34945177 10.3390/jcm10245884 PMC8707257

[22] Murabito P, Astuto M, Sanfilippo F, et al. Proactive management of intraoperative hypotension reduces biomarkers of organ injury and oxidative stress during elective non-cardiac surgery: a pilot randomized controlled trial. JCM. 2022;11:392. 10.3390/jcm11020392. 35054083 10.3390/jcm11020392 PMC8777609

[23] Frassanito L, Giuri PP, Vassalli F, et al. Hypotension prediction index guided goal directed therapy and the amount of hypotension during major gynaecologic oncologic surgery: a randomized controlled clinical trial. J Clin Monit Comput. 2023;37:1081–93. 10.1007/s10877-023-01017-1. 37119322 10.1007/s10877-023-01017-1 PMC10372133

[24] Lorente JV, Ripollés-Melchor J, Jiménez I, et al. Intraoperative hemodynamic optimization using the hypotension prediction index vs. goal-directed hemodynamic therapy during elective major abdominal surgery: the Predict-H multicenter randomized controlled trial. Front Anesthesiol. 2023;2:1193886. 10.3389/fanes.2023.1193886.

[25] Pouska J, Kletecka J, Zatloukal J, et al. A protocol based on hypotension probability indicator vs. standard care to prevent intraoperative hypotension during supratentorial brain surgery: a prospective randomized pilot trial. Minerva Anestesiol. 2023. 10.23736/S0375-9393.23.17197-5. 37158627 10.23736/S0375-9393.23.17197-5

[26] Yoshikawa Y, Maeda M, Kunigo T, et al. Effect of using hypotension prediction index versus conventional goal-directed haemodynamic management to reduce intraoperative hypotension in non-cardiac surgery: a randomised controlled trial. J Clin Anesth. 2024;93:111348. 10.1016/j.jclinane.2023.111348. 38039629 10.1016/j.jclinane.2023.111348

[27] Lai C-J, Cheng Y-J, Han Y-Y, et al. Hypotension prediction index for prevention of intraoperative hypotension in patients undergoing general anesthesia: a randomized controlled trial. Perioper Med. 2024;13:57. 10.1186/s13741-024-00414-7. 10.1186/s13741-024-00414-7 PMC11180403 38879506

[28] Li W, Hu Z, Yuan Y, et al. Effect of hypotension prediction index in the prevention of intraoperative hypotension during noncardiac surgery: a systematic review. J Clin Anesth. 2022;83:110981. 10.1016/j.jclinane.2022.110981. 36242978 10.1016/j.jclinane.2022.110981

[29] Pilakouta Depaskouale MA, Archonta SA, Katsaros DM, et al. Beyond the debut: unpacking six years of Hypotension Prediction Index software in intraoperative hypotension prevention - a systematic review and meta-analysis. J Clin Monit Comput. 2024. 10.1007/s10877-024-01202-w. 39048785 10.1007/s10877-024-01202-w

[30] Enevoldsen J, Vistisen ST. Performance of the hypotension prediction index may be overestimated due to selection bias. Anesthesiology. 2022;137:283–9. 10.1097/ALN.0000000000004320. 35984931 10.1097/ALN.0000000000004320

[31] Michard F, Futier E. Predicting intraoperative hypotension: from hope to hype and back to reality. Br J Anaesth. 2023;131:199–201. 10.1016/j.bja.2023.02.029. 36997473 10.1016/j.bja.2023.02.029

[32] Mulder MP, Harmannij-Markusse M, Donker DW, et al. Is continuous intraoperative monitoring of mean arterial pressure as good as the hypotension prediction index algorithm?: research letter. Anesthesiology. 2023;138:657–8. 10.1097/ALN.0000000000004541. 37011012 10.1097/ALN.0000000000004541

[33] Mulder MP, Harmannij-Markusse M, Fresiello L, et al. Hypotension prediction index is equally effective in predicting intraoperative hypotension during noncardiac surgery compared to a mean arterial pressure threshold: a prospective observational study. Anesthesiology. 2024. 10.1097/ALN.0000000000004990.10.1097/ALN.0000000000004990. 38558038 10.1097/ALN.0000000000004990

[34] Kouz K, Scheeren TWL, Van Den Boom T, Saugel B. Hypotension prediction Index software alarms during major noncardiac surgery: a post hoc secondary analysis of the EU-HYPROTECT registry. BJA Open. 2023;8:100232. 10.1016/j.bjao.2023.100232. 37869057 10.1016/j.bjao.2023.100232 PMC10589371

[35] Rellum SR, Noteboom SH, Van Der Ster BJP, et al. The hypotension prediction index versus mean arterial pressure in predicting intraoperative hypotension: a clinical perspective. Eur J Anaesthesiol. 2025. 10.1097/EJA.0000000000002150. 40012367 10.1097/EJA.0000000000002150 PMC12052080

[36] Ripollés-Melchor J, Tomé-Roca JL, Zorrilla-Vaca A, et al. Hemodynamic management guided by the hypotension prediction index in abdominal surgery: a multicenter randomized clinical trial. Anesthesiology. 2025;142:639–54. 10.1097/ALN.0000000000005355. 39746186 10.1097/ALN.0000000000005355

[37] Schulz KF, Altman DG, Moher D, Group C. CONSORT 2010 Statement: updated guidelines for reporting parallel group randomised trials. BMJ. 2010;340:c332–c332. 10.1136/bmj.c332. 20332509 10.1136/bmj.c332 PMC2844940

[38] Pinsky MR. Protocolized cardiovascular management based on ventricular-arterial coupling. In: Pinsky MR, Payen D, editors. Functional hemodynamic monitoring. Berlin: Springer; 2005. p. 381–95.

[39] Monge Garcia MI, Gil Cano A, Gracia Romero M. Dynamic arterial elastance to predict arterial pressure response to volume loading in preload-dependent patients. Crit Care. 2011;15:R15. 10.1186/cc9420. 21226909 10.1186/cc9420 PMC3222048

[40] Writing Committee for the VISION Study Investigators, Devereaux PJ, Biccard BM, et al. Association of postoperative high-sensitivity troponin levels with myocardial injury and 30-day mortality among patients undergoing noncardiac surgery. JAMA. 2017;317:1642. 10.1001/jama.2017.4360. 28444280 10.1001/jama.2017.4360

[41] Devereaux PJ, Chan M, Allonso-Coello P, Walsh M. Association between postoperative troponin levels and 30-day mortality among patients undergoing noncardiac surgery. JAMA. 2012;307:2295. 10.1001/jama.2012.5502. 22706835 10.1001/jama.2012.5502

[42] Walsh M, Devereaux PJ, Garg AX, et al. Relationship between intraoperative mean arterial pressure and clinical outcomes after noncardiac surgery. Anesthesiology. 2013;119:507–15. 10.1097/ALN.0b013e3182a10e26. 23835589 10.1097/ALN.0b013e3182a10e26

[43] Mehta RL, Kellum JA, Shah SV, et al. Acute kidney injury network: report of an initiative to improve outcomes in acute kidney injury. Crit Care. 2007;11:R31. 10.1186/cc5713. 17331245 10.1186/cc5713 PMC2206446

[44] Thygesen K, Alpert JS, Jaffe AS, et al. Fourth universal definition of myocardial infarction (2018). J Am Coll Cardiol. 2018;72:2231–64. 10.1016/j.jacc.2018.08.1038. 30153967 10.1016/j.jacc.2018.08.1038

[45] Marcantonio ER, Ngo LH, O’Connor M, et al. 3D-CAM: derivation and validation of a 3-minute diagnostic interview for CAM-Defined Delirium: a cross-sectional diagnostic test study. Ann Intern Med. 2014;161:554. 10.7326/M14-0865. 25329203 10.7326/M14-0865 PMC4319978

[46] Maheshwari K, Shimada T, Yang D, et al. Hypotension prediction index for prevention of hypotension during moderate- to high-risk noncardiac surgery. Anesthesiology. 2020;133:1214–22. 10.1097/ALN.0000000000003557. 32960954 10.1097/ALN.0000000000003557

[47] Grundmann CD, Wischermann JM, Fassbender P, et al. Hemodynamic monitoring with hypotension prediction Index versus arterial waveform analysis alone and incidence of perioperative hypotension. Acta Anaesthesiol Scand. 2021;65:1404–12. 10.1111/aas.13964. 34322869 10.1111/aas.13964

[48] Solares GJ, Garcia D, Monge Garcia MI, et al. Real-world outcomes of the hypotension prediction index in the management of intraoperative hypotension during non-cardiac surgery: a retrospective clinical study. J Clin Monit Comput. 2023;37:211–20. 10.1007/s10877-022-00881-7. 35653007 10.1007/s10877-022-00881-7

[49] Runge J, Graw J, Grundmann CD, et al. Hypotension prediction index and incidence of perioperative hypotension: a single-center propensity-score-matched analysis. JCM. 2023;12:5479. 10.3390/jcm12175479. 37685546 10.3390/jcm12175479 PMC10488065

[50] De Keijzer IN, Vos JJ, Yates D, et al. Impact of clinicians’ behavior, an educational intervention with mandated blood pressure and the hypotension prediction index software on intraoperative hypotension: a mixed methods study. J Clin Monit Comput. 2023. 10.1007/s10877-023-01097-z. 38112879 10.1007/s10877-023-01097-z PMC10995090

[51] Szrama J, Gradys A, Bartkowiak T, et al. The incidence of perioperative hypotension in patients undergoing major abdominal surgery with the use of arterial waveform analysis and the hypotension prediction index hemodynamic monitoring—a retrospective analysis. JPM. 2024;14:174. 10.3390/jpm14020174. 38392607 10.3390/jpm14020174 PMC10889918

[52] Wanner PM, Filipovic M. Intraoperative hypotension: new answers, but the same old questions. J Clinic Anesth. 2024. 10.1016/j.jclinane.2023.111373. 10.1016/j.jclinane.2023.111373 38191276

[53] Kouz K, Monge García MI, Cerutti E, et al. Intraoperative hypotension when using hypotension prediction index software during major noncardiac surgery: a European multicentre prospective observational registry (EU HYPROTECT). BJA Open. 2023;6:100140. 10.1016/j.bjao.2023.100140. 37588176 10.1016/j.bjao.2023.100140 PMC10430826

[54] Sessler DI, Bloomstone JA, Aronson S, et al. Perioperative Quality Initiative consensus statement on intraoperative blood pressure, risk and outcomes for elective surgery. Br J Anaesth. 2019;122:563–74. 10.1016/j.bja.2019.01.013. 30916004 10.1016/j.bja.2019.01.013

[55] Saugel B, Annecke T, Bein B, et al. Intraoperative haemodynamic monitoring and management of adults having non-cardiac surgery: guidelines of the German Society of Anaesthesiology and Intensive Care Medicine in collaboration with the German Association of the Scientific Medical Societies. J Clin Monit Comput. 2024. 10.1007/s10877-024-01132-7. 38381359 10.1007/s10877-024-01132-7 PMC11427556

[56] Sun LY, Wijeysundera DN, Tait GA, Beattie WS. Association of intraoperative hypotension with acute kidney injury after elective noncardiac surgery. Anesthesiology. 2015;123:515–23. 10.1097/ALN.0000000000000765. 26181335 10.1097/ALN.0000000000000765

[57] Wanner PM, Wulff DU, Djurdjevic M, et al. Targeting higher intraoperative blood pressures does not reduce adverse cardiovascular events following noncardiac surgery. J Am Coll Cardiol. 2021;78:1753–64. 10.1016/j.jacc.2021.08.048. 34711333 10.1016/j.jacc.2021.08.048

[58] Marcucci M, Painter TW, Conen D, et al. Hypotension-avoidance versus hypertension-avoidance strategies in noncardiac surgery: an international randomized controlled trial. Ann Intern Med. 2023;176:605–14. 10.7326/M22-3157. 37094336 10.7326/M22-3157

[59] Sessler DI, Meyhoff CS, Zimmerman NM, et al. Period-dependent Associations between Hypotension during and for four days after noncardiac surgery and a composite of myocardial infarction and death. Anesthesiology. 2018;128:317–27. 10.1097/ALN.0000000000001985. 29189290 10.1097/ALN.0000000000001985

[60] Jacquet-Lagrèze M, Larue A, Guilherme E, et al. Prediction of intraoperative hypotension from the linear extrapolation of mean arterial pressure. Eur J Anaesthesiol. 2022;39:574–81. 10.1097/EJA.0000000000001693. 35695749 10.1097/EJA.0000000000001693

[61] Vistisen ST, Enevoldsen J. CON: the hypotension prediction index is not a validated predictor of hypotension. Eur J Anaesthesiol. 2024;41:118–21. 10.1097/EJA.0000000000001939. 38085015 10.1097/EJA.0000000000001939

[62] Massari D, De Keijzer IN, Vos JJ. Comparing the hypotension prediction index to mean arterial pressure and linear extrapolated mean arterial pressure for the prediction of intraoperative hypotension: a secondary analysis. Anesthesiology. 2024. 10.1097/ALN.0000000000005198.10.1097/ALN.0000000000005198. 39377485 10.1097/ALN.0000000000005198

[63] Ranucci M, Barile L, Ambrogi F, Pistuddi V. Discrimination and calibration properties of the hypotension probability indicator during cardiac and vascular surgery. Minerva Anestesiol. 2019. 10.23736/S0375-9393.18.12620-4. 30481996 10.23736/S0375-9393.18.12620-4

