# Supplementary material for: Effectiveness of hypotension prediction index software in reducing intraoperative hypotension in prolonged prone-position spine surgery: a single-center clinical trial
Source: J Clin Monit Comput. 2025 May 23;39(5):875–87. doi: 10.1007/s10877-025-01303-0 (PMC12474604; doi:10.1007/s10877-025-01303-0)
Supplement: Supplementary file 1 — Supplementary file1 (PDF 582 KB) [file 10877_2025_1303_MOESM1_ESM.pdf]

Cumulative blood pressure parameters for MAP<50mmHg (one-tailed analysis)

	Intervention group (39)	Control Group (38)	**Hodges-Lehman estimation και 95% C.I.**	**p-value (Wilcox)**
**MAP<50mmHg**				
Total number of events	0 (0 , 0)	0 (0 , 0)	0 (-Inf , 0.001)	0.35
Average duration of each hypotensive event	0 (0 , 0)	0 (0 , 0)	-0.001 (-Inf ,0.001)	0.316
Total duration of hypotensive events per patient	0 (0 , 0)	0 (0 , 0)	-0.001 (-Inf ,0.001)	0.308
AUT MAP <50mmHg per patient	3.67 (1.67 , 5.67)	3.67 (2 , 13.33)	-1.33 (-Inf , 1.67)	0.288
TWA of AUT (MAP < 50mmHg) per patient	0.0108(0.0032,0.0162)	0.0104 (0.0051,0.0378)	-0.003(-Inf ,0.0047)	0.259

MAP, mean arterial pressure; AUT, area under threshold; TWA, time weighted average

Cumulative blood pressure parameters for MAP<50mmHg (two-tailed analysis)

	Intervention group (39)	Control Group (38)	**Hodges-Lehman estimation και 95% C.I.**	**p-value (Wilcox)**
**MAP<50mmHg**				
Total number of events	0 (0 , 0)	0 (0 , 0)	0 (-0.001 , 0.001)	0.7
Average duration of each hypotensive event	0 (0 , 0)	0 (0 , 0)	-0.001 (-0.001 ,0.001)	0.631
Total duration of hypotensive events per patient	0 (0 , 0)	0 (0 , 0)	-0.001 (-0.001 ,0.001)	0.616
AUT MAP <50mmHg per patient	3.67 (1.67 , 5.67)	3.67 (2 , 13.33)	-1.33 (-9.33 , 2)	0.575
TWA of AUT (MAP < 50mmHg) per patient	0.0108(0.0032,0.0162)	0.0104 (0.0051,0.0378)	-0.003(-0.0236,0.0063)	0.518

Effectiveness of Hypotension Prediction Index Software in Reducing Intraoperative Hypotension in Prolonged Prone-Position Spine Surgery: A Single-Center Clinical Trial

Myrto A. Pilakouta Depaskouale[1,2], MSc, Stela A. Archonta[2], MD, Moutafidou Sofia[2], MD, Nikolaos A. Paidakakos[3], MSc, Antonia N. Dimakopoulou[2], PhD, Paraskevi K. Matsota, PhD[1]

[1] 2nd Department of Anesthesiology, School of Medicine, National and Kapodistrian University of Athens, "Attikon" Hospital, Athens, Greece

[2] Department of Anesthesiology, Athens General Hospital "Georgios Gennimatas", Athens, Greece

[3] Department of Neurosurgery, Athens General Hospital "Georgios Gennimatas", Athens, Greece

Address email to myrtopde@gmail.com
