# Supplementary material for: Effectiveness of hypotension prediction index software in reducing intraoperative hypotension in prolonged prone-position spine surgery: a single-center clinical trial
Source: J Clin Monit Comput. 2025 May 23;39(5):875–87. doi: 10.1007/s10877-025-01303-0 (PMC12474604; doi:10.1007/s10877-025-01303-0)
Supplement: Supplementary file 2 — Supplementary file2 (PDF 676 KB) [file 10877_2025_1303_MOESM2_ESM.pdf]

Serum Lactate Levels at the beginning and at the end of the surgery

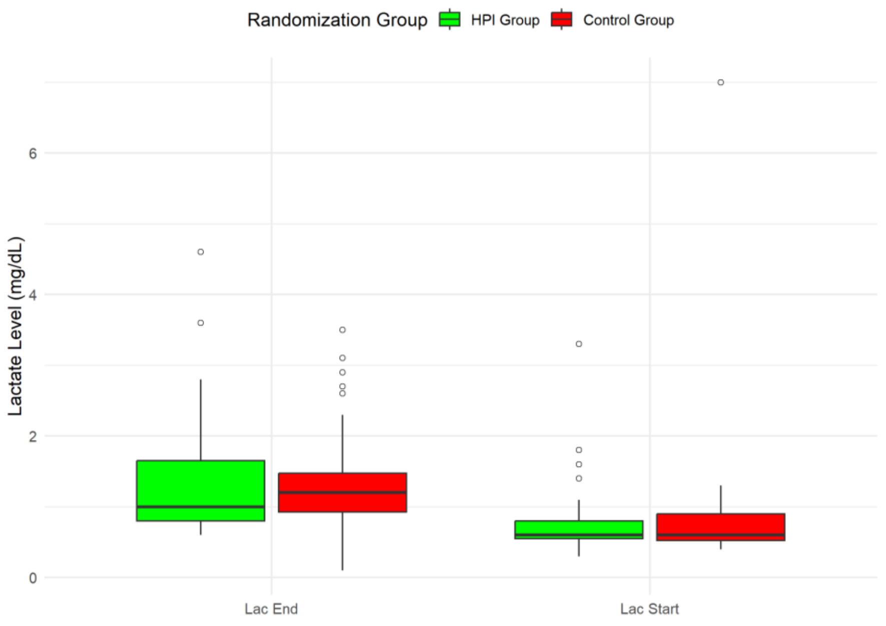

Creatinine Levels

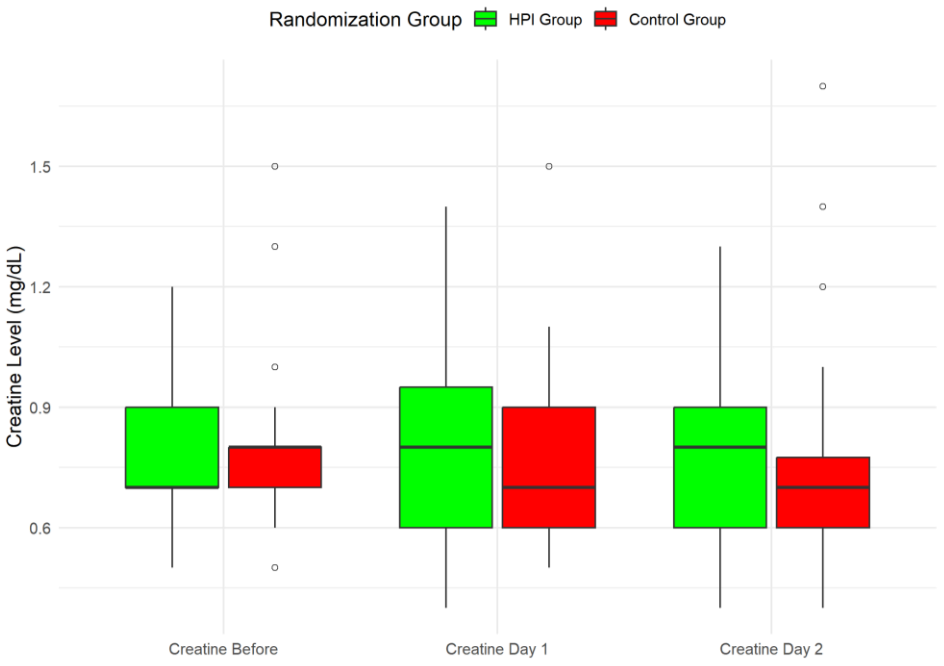

High Sensitivity Troponin I Levels

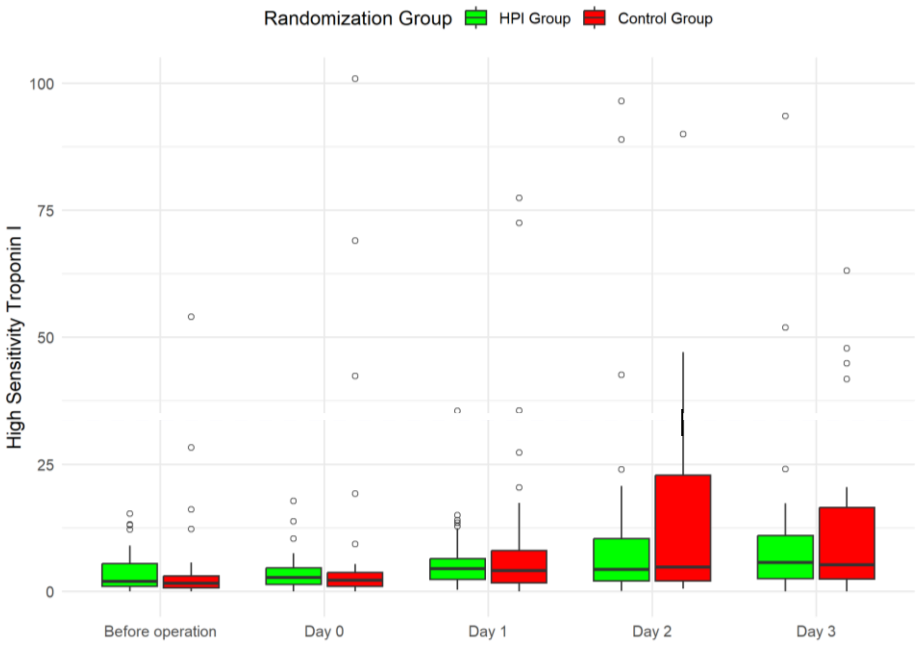

Values in pg/ml, local 99th percentile : <15.6 pg/ml

Effectiveness of Hypotension Prediction Index Software in Reducing Intraoperative Hypotension in Prolonged Prone-Position Spine Surgery: A Single-Center Clinical Trial

Myrto A. Pilakouta Depaskouale[1,2], MSc, Stela A. Archonta[2], MD, Moutafidou Sofia[2], MD, Nikolaos A. Paidakakos[3], MSc, Antonia N. Dimakopoulou[2], PhD, Paraskevi K. Matsota, PhD[1]
[1] 2nd Department of Anesthesiology, School of Medicine, National and Kapodistrian University of Athens, “Attikon” Hospital, Athens, Greece
[2] Department of Anesthesiology, Athens General Hospital “Georgios Gennimatas”, Athens, Greece
[3] Department of Neurosurgery, Athens General Hospital “Georgios Gennimatas”, Athens, Greece

Address email to myrtopde@gmail.com
