# Supplementary material for: Effectiveness of hypotension prediction index software in reducing intraoperative hypotension in prolonged prone-position spine surgery: a single-center clinical trial
Source: J Clin Monit Comput. 2025 May 23;39(5):875–87. doi: 10.1007/s10877-025-01303-0 (PMC12474604; doi:10.1007/s10877-025-01303-0)
Supplement: Supplementary file 3 — Supplementary file3 (PDF 276 KB) [file 10877_2025_1303_MOESM3_ESM.pdf]

<u>Cumulative blood pressure parameters: Two-sided testing</u>

	Intervention group(39) Median (IQR)	Control Group (38) Median (IQR)	Hodges-Lehman estimation (95% C.I.)	p-value (Wilcox)
**MAP<65mmHg (primary outcome)**				
Total number of hypotensive events	2 (0.5 , 5)	4 (1 , 7)	-1 (-3 , 0.001)	0.060
Average duration of each hypotensive event(min)	2 (0.5 , 3.42)	2.385 (1.68 , 3.26)	-0.38 (-1.24 , 0.19)	0.191
Total duration of hypotensive events per patient(min)	4 (0.5 , 12.17)	11.17 (2.58 , 20.08)	-4 (-9 , -1.45)	**0.038**
Mean MAP <65mmHg per patient (mmHg)	59.71 (58.2, 60.62)	60.61 (59.71 , 61.37)	-0.86(-1.82, 0.2)	0.115
AUT MAP< 65mmHg for per patient	47.33 (24 , 98.33)	64.33 (32.67,114.33)	-13.33(-44, 14)	0.262
TWA of AUT (MAP < 65mmHg) per patient (mmHg)	0.1 (0.05, 0.23)	0.145 (0.088, 0.368)	-0.04(-0.12, 0.02)	0.176
**Postinduction MAP<65mmHg**				
Total number of hypotensive events	1 (0 , 1.5)	1 (0.25 , 2)	-0.001 (-1 , 0.001)	0.051
Average duration of each hypotensive event (min)	1 (0 , 2.34)	2.25 (0.25 , 3.82)	-1 (-2 , -0.001)	**0.024**
Total duration of hypotensive events per patient (min)	1 (0 , 3.84)	4 (0.25 , 5.67)	-1.33 (-3.67, -0.001)	**0.021**
Mean MAP < 65mmHg per patient (mmHg)	59.33 (58.16 , 61.4)	59.32 (57.73, 60.99)	0.01 (-1.62 ,1.64)	0.989
AUT MAP< 65mmHg for per patient	22.85 (8.92, 50.67)	27.67(13.58 ,53.5)	-4.15 (-19.33,10.66)	0.558
TWA of AUT (MAP < 65mmHg) per patient (mmHg)	1.14 (0.27, 2.06)	1.34 (0.65,2.33)	-0.23 (- 0.83 , 0.5)	0.579
**Operative Time (First Incision to Last Suture) MAP<65mmHg**				
Total number of hypotensive events	0 (0 , 2)	0 (0 , 4)	-0.001 (-1 , 0.001)	0.194
Average duration of each hypotensive event (min)	0 (0 , 1.54)	0 (0 , 2.05)	-0.001 (-0.001 , 0.001)	0.373
Total duration of hypotensive events per patient (min)	0 (0 , 3.67)	0 (0 , 11.25)	-0.001 (-1 , 0.001)	0.204
Mean MAP < 65mmHg per patient (mmHg)	60.42 (0 , 59.07)	61.91 (0 , 61.73)	-0.66 (-2.57, 0.68)	0.262
AUT MAP< 65mmHg for per patient	26.17 (15.92 ,55.25)	37.33 (28 ,74.33)	-13.34 (-0.67,10.34)	0.292
TWA of AUT (MAP < 65mmHg) per patient (mmHg)	0.075 (0.033,0.165)	0.12 (0.08 , 0.36)	-0.05 (-0.14, 0.02)	0.262

Effectiveness of Hypotension Prediction Index Software in Reducing Intraoperative Hypotension in Prolonged Prone-Position Spine Surgery: A Single-Center Clinical Trial

Myrto A. Pilakouta Depaskouale[1,2], MSc, Stela A. Archonta[2], MD, Moutafidou Sofia[2], MD, Nikolaos A. Paidakakos[3], MSc, Antonia N. Dimakopoulou[2], PhD, Paraskevi K. Matsota, PhD[1]

[1] 2nd Department of Anesthesiology, School of Medicine, National and Kapodistrian University of Athens, "Attikon" Hospital, Athens, Greece

[2] Department of Anesthesiology, Athens General Hospital "Georgios Gennimatas", Athens, Greece

[3] Department of Neurosurgery, Athens General Hospital "Georgios Gennimatas", Athens, Greece

Address email to myrtopde@gmail.com
